# DETERMINING THE ASSOCIATION BETWEEN THE 24 HOUR COMPOSITION OF WAKING BEHAVIORS AND SLEEP WITH RECURRENT FALLS

**DOI:** 10.1093/geroni/igad104.2785

**Published:** 2023-12-21

**Authors:** Lauren Roe, Elsa Strotmeyer, Peggy Cawthon, Nancy Glynn, Kristine Ensrud, Katie Stone, Kelley Pettee Gabriel, Jane Cauley

**Affiliations:** University of Pittsburgh, Pittsburgh, Pennsylvania, United States; University of Pittsburgh, Pittsburgh, Pennsylvania, United States; California Pacific Medical Center, Research Institute, San Francisco, California, United States; University of Pittsburgh School of Public Health, Pittsburgh, Pennsylvania, United States; University of Minnesota, Minneapolis, Minnesota, United States; California Pacific Medical Center, San Francisco, California, United States; School of Public Health, Birmingham, Alabama, United States; University of Pittsburgh, Pittsburgh, Pennsylvania, United States

## Abstract

Physical activity (PA), sleep, and sedentary behavior are individually associated with falls, but analyses examining one activity omit information about all other activities. We examined the combination of PA, sedentary behavior, and sleep on recurrent fall risk (2+ falls/year in any year) in older men using compositional data analysis. Data were from 2,705 men aged 79.0±5.1 years in the Osteoporotic Fractures in Men Study (MrOS). Twenty-four-hour accelerometer-measured time sedentary (< 1.5 METs), physically active (PA; >1.5 METs), and sleeping were used to calculate isometric logratios (ILR). ILRs were predictors in logistic regression models estimating the proportion of time in one activity relative to time in all other activities simultaneously, associated with recurrent falls. A total of 1,025 (37.9%) men experienced recurrent falls after four years of follow-up. In minimally adjusted models (age, race, clinic site, season of accelerometer wear), more PA relative to sedentary behaviors and sleep equated to lower odds of recurrent falls (OR: 0.70, 95%CI: 0.60, 0.79). More sedentary behavior relative to PA and sleep resulted in higher odds of recurrent falls (OR: 1.54, 95%CI: 1.14, 2.09). Associations were substantially attenuated after adjustment for height, weight, diabetes, health and smoking status, comorbidities, and instrumental activities of daily living impairments (MVPA vs. sedentary and sleep – OR: 0.88, 95%CI: 0.75, 1.02; sedentary vs. PA and sleep – OR: 1.33, 95%CI: 0.97, 1.82) and not significant. More PA and less sedentary behavior relative to other behaviors were associated with recurrent falls, with associations explained by poor health and comorbidities.

